# Pathological Anatomy

**Published:** 1860-07

**Authors:** John H. Packard

**Affiliations:** Philadelphia


### PATHOLOGICAL ANATOMY.

BY JOHN H. PACKARD, M.D., OF PHILADELPHIA.

I.—*Pathology of the Pituitary Body.* By MIDDLETON MICHEL, M.D., of Charleston, S. C.

A negro carpenter, aged thirty-one, of robust frame and steady habits, was first affected with headache and weakness of sight in September, 1851. The headache became intermittent, the imperfection of sight progressed steadily; the eyes started forward, and his appearance was that of an amaurotic person. Ptosis, and an extreme degree of chemosis occurred soon after he came under Dr. Michel's care, in March, 1855. A severe attack of mental excitement came on about the end of April, but lasted only a few days. Soon after this a tumor appeared in the right temporal region, with deafness on that side; his smell was lost, and his taste perverted. Memory and the co-ordination of his muscular powers were evidently impaired. An obstinate diarrhœa of eleven days continuance carried him off in September, 1855.

An autopsy showed a tumor of the size, form, color, and consistence of a ripe blue fig, occupying the place of the pituitary body; a much larger mass, attached to this by a pedicle, extended outward to the right temporal fossa. The base of the skull was much disorganized. "The nerves were found but little influenced by the disease around them, and though not changed in structure singularly reduced in size; among other circumstances noticed, the optic nerves were mere threads and pressed perfectly flat, the pons and the medulla oblongata were so far atrophied as not to exceed the little finger in size"

No satisfactory microscopic examination was made of this growth, but Dr. Michel regarded it as undoubtedly cancerous.

The case now sketched is made the basis of a paper on the pathology of the pituitary body. Atrophy has been observed in one instance by the author, and is mentioned by Morgagni, Wenzel, and others. Hypertrophy is believed by Lebert to be sometimes mistaken for degeneration; its occurrence is denied by Rokitansky, although Wepfer, Chaussier, and Rayer record instances of it. Inflammation of this organ, and cysts in connection with it, are described by Andral, Delasiauve, Zenker, and others. Adventitious deposits, sabulous and calcareous, have been observed by some. Cancer, in all its varieties, may affect the pituitary body; Dr. Michel's case was one of encephaloid, and for this as well as the other forms we have the authority of Vieussens, De Haen, Levêque-Lasource, Abercrombie, Vidal, and Rokitansky. The author concludes his paper with the following propositions:—

(1) "The pituitary body, however largely developed in some animals, is not a primary division of the brain, or a true encephalic ganglion, since its complete destruction is never accompanied by loss of intellection, motion or sensation, beyond what may be satisfactorily accounted for by the necessary pressure which the morbid growth exerts upon more essential parts of the encephalon.

(2) "From several of the morbid processes enumerated in this memoir, we have strong proof of the identity of the nature of this hypophysis with certain so-called vascular glands, such as the thyroid, thymus, spleen, and supra-renal capsules.

(3) "While the diagnosis of its morbid conditions is rendered somewhat obscure from the absence of any ascertained function of the part, yet their almost constant connection with the simultaneous production of amaurosis in both eyes, with absence of symptoms of cross paralysis, will indicate the seat of the disease, when compared to morbid states of either hemisphere.

(4) "The long continuance of disease in this situation may propagate inflammatory action to neighboring parts followed by apathy, somnolency, syncope, cophosis, and other symptoms obscuring the diagnosis."—(*Charleston Med. Jour. and Review*, March, 1860.)

### II.—*Medullary Fungus of the Pineal Gland, with relatively trifling Phenomena during Life.*

A laborer, aged thirty-five, having been struck on the head with a hammer, became melancholy, and suffered from periodical headache; he then had some violent illness, in the course of which an abscess formed just below the right clavicle, and was twice opened, an enormous quantity of pus being let out each time. After his recovery, signs of cerebral disease appeared, chiefly incomplete general paralysis and mental confusion. Death took place eighteen months after the infliction of the blow upon the left temple.

"*Dissection twenty-four hours after death.*—The body in general presented no phenomena worthy of mention. After removing the galea aponeurotica, however, the bones of the skull, particularly the ossa bregmatis, exhibited an unusually dark color, tending to red. The dura mater was tolerably firmly adherent to the cranium. Its arteries and veins were, on the surface, very highly congested, and the blood, on opening the sinus, flowed out of a dark-red color. The convolutions of the hemisphere were much flattened and extremely dry (which gave them the appearance of wax casts,) without the slightest trace of serum between the arachnoid and pia mater; in the cavity of the spinal column, however, there was a very large quantity of spinal fluid, which washed the medulla oblongata; the glandulæ Pacchioni on the surface were not unusually developed. The pia mater was difficult of separation, particularly in the middle and superior part, but did not remove any laminæ of the brain with it. There was no trace of meningitis. The cortical substance, which everywhere presented a rather high color, particularly on the anterior lobe, under the os frontis, after washing with water, was somewhat more of a rose color than the other parts, while the white substance exhibited none of the well-known dots of blood. The cerebral vessels on the surface contained but little blood. On cutting down into the ventricles it appeared that the pia mater investing them was very much thickened, so that the white cerebral substance could easily be separated from it, and it required some pains to cut through it. On opening the right rather dilated ventricle, whence a considerable quantity of clear serum flowed, we came on a rather long vesicle, studded with some dark-brown vesicles, immediately under the fornix and great commissure, in the situation of the foramen of Monro, which here appeared to be largely dilated; the length of the vesicle, from before backward, was five and a half centimetres (a little more than two inches;) on the right side the corpus striatum and thalamus were apparently healthy, somewhat pushed to the side, the walls of the vesicle passed uninterruptedly into the walls of the ventricles, so that it appeared that the pia mater which lines these cavities internally also invested the vesicle.

"At the level of the great commissure the cerebral mass expanding over the left ventricle appeared to be much more swollen; the investing membrane was here much denser and thicker, and could not be torn without difficulty. On opening the ventricle much bloody serum escaped, contrasting with the right cavity, where the serum was clear. The cavity of the anterior left cornu was about as large as a duck-egg, and was filled with recent coagulated blood. It now appeared that the vesicles in the right cavity were connected with those in the left, as they were full before the opening of the left ventricle, but collapsed after it. About in the situation of the thalamus under the fornix was a bloody, yellow, soft substance, presenting the appearance of medullary fungus colored with blood. The vesicle in the right cavity was covered with a membrane composing the lateral wall of the septum lucidum and passing into the investing membrane of the ventricle; so that also anteriorly the wall of the vesicle passed into the investing membrane of the cornu anticum.

"The left ventricle was, as has already been mentioned, much more distended, owing to the presence of an abnormal, medullary body; it was partly filled with coagulated blood, which was also separately effused in several parts of the tissue; on the left side, the sac extended into the cornu posticum, to the commencement of the calcar avis, covered by an organized membrane; which again was connected with the walls of the ventricles, and was uninterruptedly continued into them; on accurate examination, after the brain had been a couple of days left to harden in spirit, it was seen, by lifting up the posterior portion of the corpus callosum, that the abnormal body was continued backward through the very dilated third ventricle, and here terminated, just in front of the corpora quadrigemina, which were more or less flattened, and that the tumor consisted of a degeneration of the pineal gland. This was reduced to a matter of certainty by the microscopic examination made by Professors Schrœder van der Kolk and Harting; thus in the tumor, even on a level with the corpora striata. the peculiar conglomerations were found constituting the so-called cerebral sand, which was here present in great quantity as mulberry-like granules, and is distinguished from the sand in the choroid plexuses, which latter always exhibits spherical globules. The choroid plexus in each ventricle was normal and was not connected with the tumor; the tumor itself appeared under the microscope to consist chiefly of granular cells the size of blood corpuscles, from which, however, they were plainly distinguished by their granular contents, their unequal and mostly oblong form, without any defined nucleus; these cells were everywhere, both on the surface of the investing membrane and within the tumor, exactly similar, and between them were capillary vessels arranged in a reticulated form, and connective tissue—nerve-fibres were not to be distinguished with certainty. Hence it follows that the tumor consisted of a cellular augmentation of the pineal gland, which must be regarded as medullary fungus with sanguineous effusion, and therefore also as fungus hæmatodes. The cerebellum was healthy, the posterior commissure was normal, the cavity in which the pineal gland is situated was, in this instance, invested as with a membrane, and the third ventricle was very wide, being dilated to twenty-six millimetres (a little more than an inch.) Above the corpora quadrigemina there was also coagulated blood, the corpora quadrigemina were somewhat flattened, the enlarged pineal gland thus seemed to have pushed forward, to have dilated the third ventricle to an extraordinary degree, and thus to have pressed into the anterior cornua of the lateral ventricles, particularly on the left side, without having produced any other degeneration of the brain than was caused by pressure and distention."

In this case there was no hemiplegia or complete paraplegia, because the corpora striata and the thalami were uninjured; no abnormity in the face, eyes, or pupils, because the tumor was above the corpora quadrigemina; no decided paralysis, because of the slow development of the morbid growth. Death was brought on by hemorrhage from the tumor into the left ventricle.—(Translated from the *Geneeskundige Courant*, [Dutch,] Dec. 25, 1859, for the *Dublin Med. Press*, March 28, 1860, by William D. Moore, M.B., etc.)

III.—*On Contraction and Obliteration of the Aorta near the Junction of the Ductus Arteriosus.* By THOMAS B. PEACOCK, M.D., etc.

In this very interesting paper, 40 published cases are given in abstract, and the following is the substance of the author's concluding remarks:—

In 29 of the cases, the stricture was at or below the point named; in 4, there were two strictures. The closure of the canal was complete in 10 cases, partial in the remaining 30. Dilatation and disease of the cardiac portion of the vessel existed in 10 cases, dilatation alone in 11.

The collateral circulation is carried on by means of the branches of the subclavian and of the aorta below the seat of constriction, on both sides. (1) The transversalis colli, coming from the thyroid axis, anastomoses by its posterior scapular branch with the posterior branches of the aortic intercostals. (2) The superior intercostal, coming from the subclavian, anastomoses with the anterior branches of the aortic intercostals. (3) The internal mammary, derived also directly from the subclavian, and the external thoracic, from the axillary, anastomoses with the anterior aortic intercostals, the external epigastric, etc.

Since, however, these channels are less free than the normal aorta would be, the heart is affected as in any other form of aortic obstruction. In 10 cases there was a moderate amount of hypertrophy and dilatation involving only or chiefly the left ventricle; in 18, the enlargement was general and extensive; in 2 only, the heart is said to have been quite healthy.

Of 38 cases in which the age is reported, 23 were between twenty-one and fifty years; the extremes were twenty-two days and ninety-two years. As to sex, 30 were males and 10 females.

Death was caused by the condition of the aorta in 8 cases, and probably in another where apoplexy was the assigned reason. In 11 cases the patients succumbed to diseases which were at least but indirectly connected with the obstruction; in 16, to cardiac asthma and dropsy, variously complicated.

The origin of the abnormity is probably congenital; but the theory of its production has been variously explained: (1) by the prolongation into the aorta of a thrombus formed in the closure of the ductus arteriosus; (2) by the propagation of the process of obliteration from the latter vessel to the former; (3) by defective development of the portions of the bronchial arches which form the continuation of the aorta from the origin of the left subclavian to below the entrance of the ductus arteriosus. This latter view would seem to be the correct one.

Cases sometimes occur in which the descending aorta is traversed only by blood derived from the pulmonary artery, its communication with the ascending portion and arch being cut off.—(*Brit. and For. Med.-Chirurg. Review*, April, 1860.)

[A tabular statement of the cases mentioned in Dr. Wilks's paper will be found in the abstracts relating to the "Practice of Medicine," by Dr. R. J. Dunglison.]

### IV.—*Hydatid Tumor in the Apex of the Right Ventricle of the Heart.* By DR. GEORGE BUDD.

A single woman, aged twenty-three, stout and florid, but troubled for several years with cough and dyspnœa, was admitted into King's College Hospital, December 23, 1857. Her feet were somewhat œdematous, and there was a systolic rasping sound over the base of the heart, which however disappeared in a few days, and was not heard again. She had several hemorrhages at various intervals, and occasionally complained of intense præcordial pain. Death, which took place on the 4th of May, 1858, was preceded by œdema of the legs, ascites, and great dyspnœa.

"*Post-mortem examination.*—Both lungs were found united to the pleura costalis by old adhesions. The pericardium contained about an ounce of serous fluid. Its layers posteriorly were glued together by tolerably old adhesions. The heart was of very irregular shape, flattened anteriorly and bulging posteriorly. Its irregular shape was owing to a hydatid tumor, about the size of an orange, situated in the apex of the right ventricle, and projecting into its cavity. The right auricle and ventricle were filled with clotted blood; the left chambers of the heart were healthy. There was no disease of the valves.

"Under one of the laminæ of the tricuspid valve, a small flaccid hydatid was found unattached. In the pulmonary artery, immediately above the valves, an unbroken hydatid, rather more than half an inch in diameter, was found; and in the further course of the artery, before its subdivision, there were several other smaller hydatids.

"On tracing the branches of the pulmonary artery, several clusters of hydatids, and the collapsed skins of hydatids—ranging from one-eighth to one-fourth of an inch in diameter—were discovered in them. These hydatids were exclusively confined to the left lung, and chiefly to the upper lobe, one small cluster only being found in the centre of the lung, and one in the lower lobe. These clusters of hydatids were enveloped in pale fibrin, but not contained in organized sacs. The lower lobes of both lungs were carnified, but still crepitated slightly under the fingers. The pulmonary veins and the bronchial tubes contained no hydatids.

"The abdominal organs and the brain, and the principal venous trunks of the lower extremities, were next carefully examined; but with the exception of slight fatty degeneration of the liver, some irregularity of the surface of the right kidney, and general venous congestion, nothing abnormal was detected.

"On examining one of the smaller hydatids taken from the pulmonary artery, I found it to contain very perfect echinococci.

"The hydatid tumor in the apex of the heart was stuffed with hydatids, and it was evident that the hydatids found in the right ventricle and in the pulmonary artery had escaped from it."—(*Transactions of Pathological Society of London*, vol. x. p. 80.)

### V.—*Abscess in the Heart.*

M. Lancereaux recently presented to the *Société de Biologie* the heart of a young man, aged twenty-five, within the walls of which an abscess had developed itself. Continued fever, with very marked exacerbations, had set in toward the last, and as the patient had been subject to intermittent fever eight years previously, and his spleen was enlarged, sulphate of quinia was given, but without success; arsenic likewise failed. Auscultation disclosed a *bruit de souffle*, but no one suspected any organic disease. The autopsy showed a swelling, on a line with the valves, [it is not stated *which* valves,] as large as a filbert. When this tumor was cut into, a puruloid liquid escaped, which under the microscope exhibited blood corpuscles and pyoid bodies. These last were rounded and finely granular, but without well-formed nuclei. All the other organs were examined with care, but there was no evidence of any abscess, or of phlebitis.—(*Journal du Progrès*, April 13, 1860.)

VI.—*Hydatid Tumor and Abscess of the Liver.* By A. D. GULLAND, ESQ., M.D., Assistant Surgeon, Indian Army.

A powerful man, aged twenty-five, a gunner, temperate in his habits, was admitted to hospital on June 21st, 1859, with pleurisy of the left side. A painless enlargement of the hypochondriac and epigastric regions, which existed on his admission, increased steadily; on the 4th of July he had a good deal of retching and vomiting, followed by fever, which assumed a hectic type, and carried him off on the 8th.

At the autopsy, the abdomen presented the following appearances: "Liver pushed into the left hypochondrium, and projected several inches below the ensiform cartilage, by a large tumor, about the size and shape of a distended stomach, firmly adherent to its right lobe at its outer border, and occupying the right hypochondriac region. In removing the liver an abscess burst, discharging about a pint of very fetid blackish pus. Weight of the liver, with tumor attached, was ten pounds; without tumor, six pounds. No other adhesions of liver. Two abscesses found in the left lobe—one very large, the other small; and a third of considerable size, in the lobulus quadratus, filled with the same kind of fetid pus. The remaining portion of the left lobe was red and mottled; the right lobe of a grayish color; gall-bladder nearly empty. The tumor, when dissected from the liver, weighed four pounds. It proved to be a large hydatid sac, filled with numerous hydatids—upwards of two hundred—varying in size from a pea to a large orange. The majority of the contained cysts had burst, so that the containing cyst was partly filled with a quantity of thin membranous shreds floating in a liquid. The liquid in the unbroken cysts, of which there were sixty, was pure, transparent, and colorless. The coat of the containing sac was fibrous and thick." Other organs healthy.—(*Edinburgh Medical Journal*, March, 1860.)

VII.—*Rupture of the Spleen from a Trifling Cause.* This case was recently communicated to the Surgical Society of Ireland, by A. LEITH ADAMS, M.B., Surgeon to the 22d Regiment.

In October, 1853, a Sepoy, who was in hospital for intermittent fever, went on his well day to see a friend; but "when he had seated himself on the floor, cross-legged, (Oriental fashion,) he became suddenly faint, and expired before medical aid could be obtained. About half an hour elapsed between the first appearance of syncope and death." The man was greatly emaciated.

"When the abdomen was opened, a mass of coagulated blood, fully an inch in thickness, covered the anterior surface of the cavity, extending upward into the spleen, which, although shriveled and contracted, was still much enlarged. * * * On its lower surface there was a transverse fissure three inches in length, and nearly one inch in depth, to which the coagulum was attached. About a pint of serous fluid was found in the abdominal cavity. The substance of the spleen was very soft, almost pultaceous; the pancreas congested and enlarged; the liver pale and anæmic, which was the general appearance of the whole body, with the exception of the above organs; it was as though his life-blood had been concentrated in the spleen and pancreas only. * * * * Every circumstance of the case tended to show that the rupture took place about the time he was seating himself, when the enlarged spleen was subjected to compression consequent on posture and the action of the abdominal muscles. I could imagine a like accident occurring when the patient is straining at stool."—(*Dublin Medical Press*, March 21, 1860.)

VIII.—*General Phlegmon of the Abdomen.* By M. PETITHAN.

This affection is rare, except as met with in the puerperal state; the following case is remarkable also from the extent to which the suppurative action took place.

A gunner, aged twenty, having alighted with great force upon the soles of his feet, sustained a severe shock, and great pain about the navel. Four days afterwards, symptoms of peritonitis were developed; the inflammation extended downward to the left testicle, in the coverings of which an abscess formed. Obscure fluctuation was perceptible over the abdomen, with pain, not very severe, but extending up to the left shoulder. On the ninth day the man died.

Upon post-mortem examination, all the cellular tissue around the viscera, beneath the peritoneum, and between the muscular layers of the abdominal walls, was found replaced by pus. The inflammation had extended to the scrotum and lower extremities by involving the tissue closing the different orifices. * * * * The psoas, iliacus, and quadratus lumborum muscles on each side, as well as the cortical substance of the kidneys, were somewhat broken down. It is singular that in the midst of such extensive mischief, the peritoneum bore only a very secondary part; there was no effusion into its cavity, but merely a few adhesions at the foramen of Winslow, and some scattered patches of redness.—(*Gaz. Hebdomadaire*, March 23, 1860, from *Arch. Belges de Méd. Milit.*, October, 1859.)

IX.—*Cases of Renal Abnormity.*

1. *Enormous Abscess of the Kidney.* By DR. JACOB HAYES, of Charlestown, Mass.—The patient, a man aged thirty-three, had the usual symptoms of typhoid fever; a large tumor developed itself in the abdomen, somewhat to the right side, and at the fifth week began to soften, with the constitutional indications of suppuration. In the seventh week, it was punctured, and two quarts of pus were evacuated. For two weeks the discharge was copious, but it then became less. Purulent expectoration was now observed, and in three weeks from the time of the operation the man died, exhausted. "At the post-mortem examination, a large quantity of pus was found in the cavity of the abdomen, and the intestines were glued together with soft lymph, showing recent peritoneal inflammation. All the abdominal organs were healthy except the right kidney, which, by its dilatation, formed the tumor; this consisting of a large sac with thin walls, containing pus, and evidently formed by an expansion of the kidney. The sac was nowhere adherent to the contiguous peritoneal surface. It extended upward to the diaphragm, which was perforated, allowing the pus to pass into the right side of the chest. The right lung lay in contact with the spine, was entirely deprived of air, and quite friable. No communication with the pleural cavity was noticed[?], but attention was not directed to this point. The chest and abdomen contained six quarts of pus."—(*Boston Med. and Surg. Journal*, March 8, 1860.)

2. *Tumor of the Kidney, communicating with the Intestine.* By DR. CABOT.—A married woman, aged thirty, felt a tumor as big as a walnut in the right iliac region, just after the birth of a still-born child. Three months after, the tumor had grown so as to extend up into the hypochondrium. Its upper part was firm, the lower fluctuating; it seemed attached above, but free below. Six weeks after this, the tumor became less, and pus was noticed in the alvine discharges. Œdema, severe pain in the back, and purulent diarrhœa, preceded death, which ensued about six weeks later.

Autopsy, by DR. ELLIS.—"The ascending colon, and that part of the small intestine just below the duodenum, adhered to a firm tumor, which lay in the right lumbar region. Two inches above the superior spinous process of the right ilium was an opening upon the anterior face of the tumor, from which pus issued. Above this, the abdominal parietes had evidently been slightly adherent, and were sufficiently eroded and discolored to show that the pus was seeking an outlet in that direction. Behind the tumor was a large collection of pus, which extended downward, beneath the lumbar and iliac muscles. One-half of the lower dorsal, and the whole of the upper lumbar vertebræ were denuded. On incision the remains of the kidney were found within the thickened tissues. The organ was of about the usual size, mostly occupied by cavities, the largest of which was an inch and a half in diameter. They were filled with thick pus. The parietes were of a dark-bluish slate color, and plicated. In that portion of the large intestine which adhered to the kidney was a small opening, through which a probe could be passed into one of the large cavities. A free communication was also established with the small intestine at the point designated; but here were two openings each half an inch in diameter. The mucous membrane of the part was of a grayish color. The remaining substance of the kidney, between the cavities, was firm, uniform, and grayish, but presented no appearance of the usual structure. Left kidney was large, yellowish and 'coarse.'" (*Ibid.*)

3. *Absence of the Bladder; Enlargement of the Pelvis of the Kidney.*—M. Schmidt states, in the *Jour. de Méd. de Bruxelles*, that a woman, aged thirty, died at the Central Hospital of the Grand Duchy of Luxembourg, who presented, on a post-mortem examination, a complete absence of the bladder. The right kidney was very large, and its pelvis so increased in size that it could contain four or five ounces of fluid. It had evidently performed the office of a bladder. It was terminated by a very long ureter, which opened at the meatus. The left kidney was quite atrophied, and seemed to be affected with tubercular degeneration. The woman had stated that she had suffered from incontinence of urine since her twelfth year—a circumstance which can hardly be credited, when the congenital defect is considered.—(*London Lancet*, March 31, 1860.)

